# In memoriam of Pawel P. Liberski (1954–2025)

**DOI:** 10.1080/19336896.2026.2640273

**Published:** 2026-03-05

**Authors:** Beata Sikorska

**Affiliations:** a Department of Molecular Pathology and Neuropathology, Medical University of Lodz, Lodz, Poland; b Reference Centre for Prion Diseases, Lodz, Poland; c Department of Pathomorphology, University Hospital in Krakow, Krakow, Poland; d The Mazovian Academy in Plock, Plock, Poland

**Keywords:** In memoriam, Pawel Liberski, prion diseases, researchers

## Abstract

In memoriam of Pawel P. Liberski, an enthusiastic scientist of rare intelligence, a loyal and generous friend, and a truly vivid personality.

19 August 2025, will remain in my memory as the sad day I lost my mentor and friend, Pawel P. Liberski. Pawel was an enthusiastic scientist of rare intelligence, a loyal friend, and a truly vivid personality. We all remember his colourful blazers, red suspenders, his fondness for cigars and good food – and his distinctive sense of humour. With his passing, we lost a colleague of remarkable intellect and charisma.

Pawel Liberski (Figures 1, 2 , and 3). was born on 25 November 1954, in Zgierz near Lodz, Poland. Not everyone may know that he came from an artistic family. Both of his parents were painters, and his father’s works can still be seen in Polish art museums That background likely had a strong influence on Pawel’s character and his lifelong interest in art. He collected art, and his offices – both at home and at work – felt like small museums (Figure 4). He gathered prints and Japanese woodblock works, and above all, art from Indigenous peoples of Oceania, Asia, and Africa. Alongside art, he collected rare books. He read widely, was a true connoisseur of literature, and often quoted Shakespeare as readily as Tolkien, Churchill, or Bulgakov. He was also interested in history, battleships and Japanese culture. He was a true Renaissance man. We all knew that if Pawel was missing from the lecture hall during a conference, he was most likely exploring a museum somewhere nearby.

**Figure 1.**
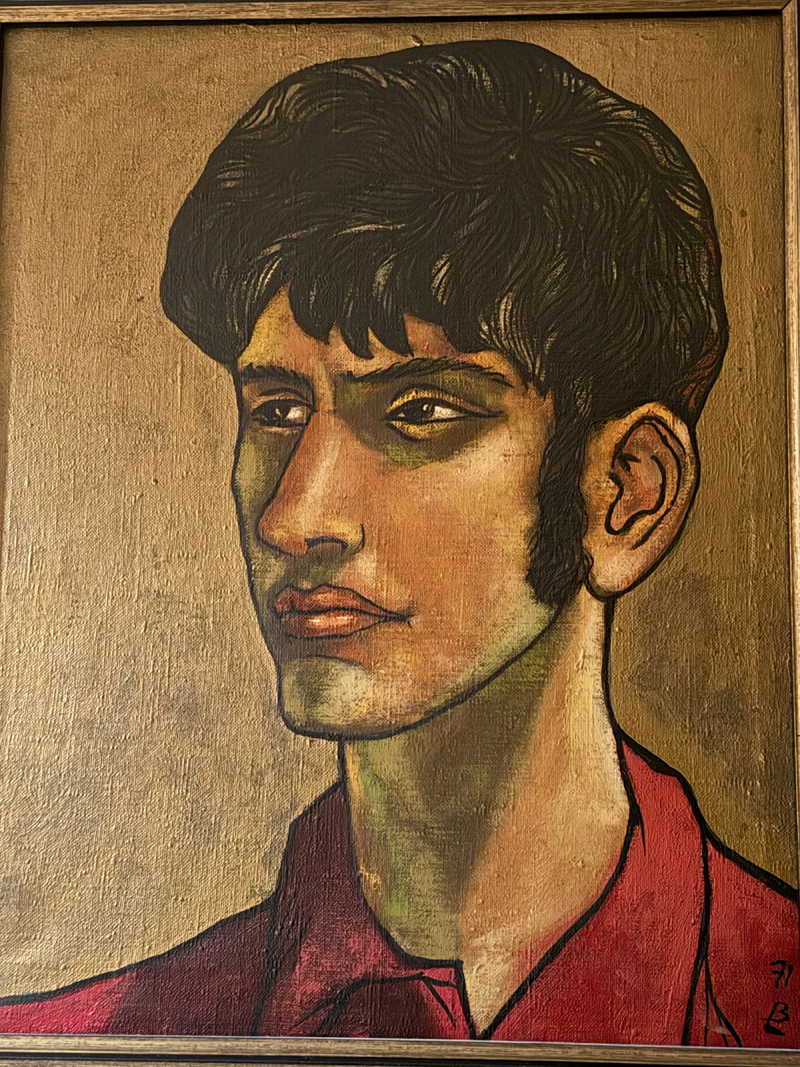
Pawel Liberski at the age of 17, painted by his father Benon Liberski (courtesy of Professor Maria Respondek-Liberska).

**Figure 2.**
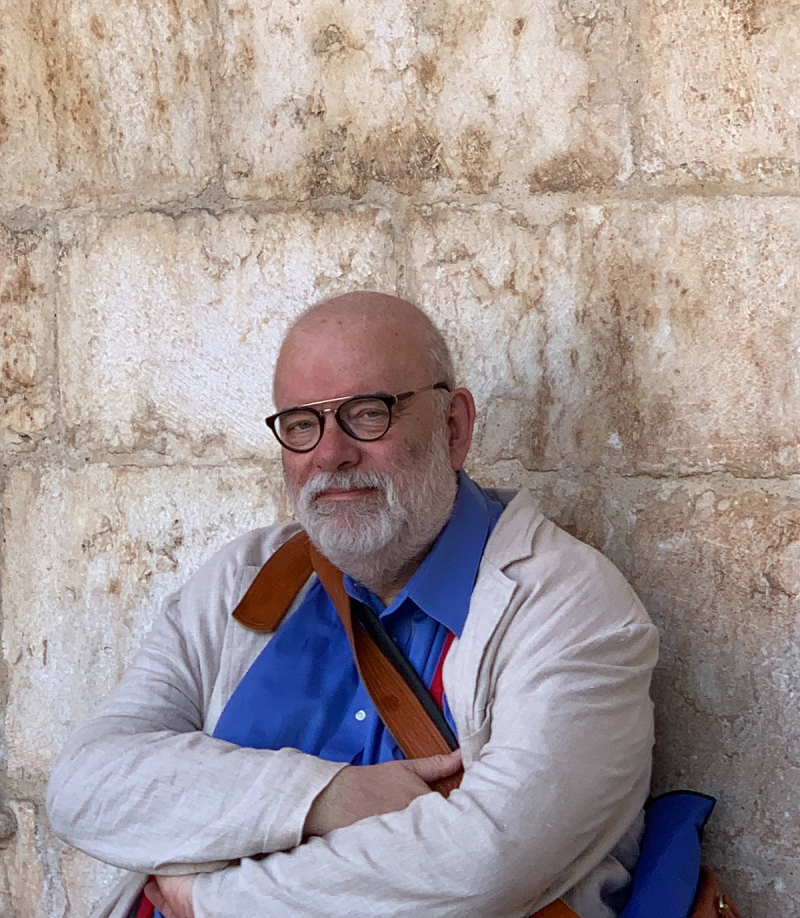
Pawel Liberski in Lisbon, 2019.

**Figure 3.**
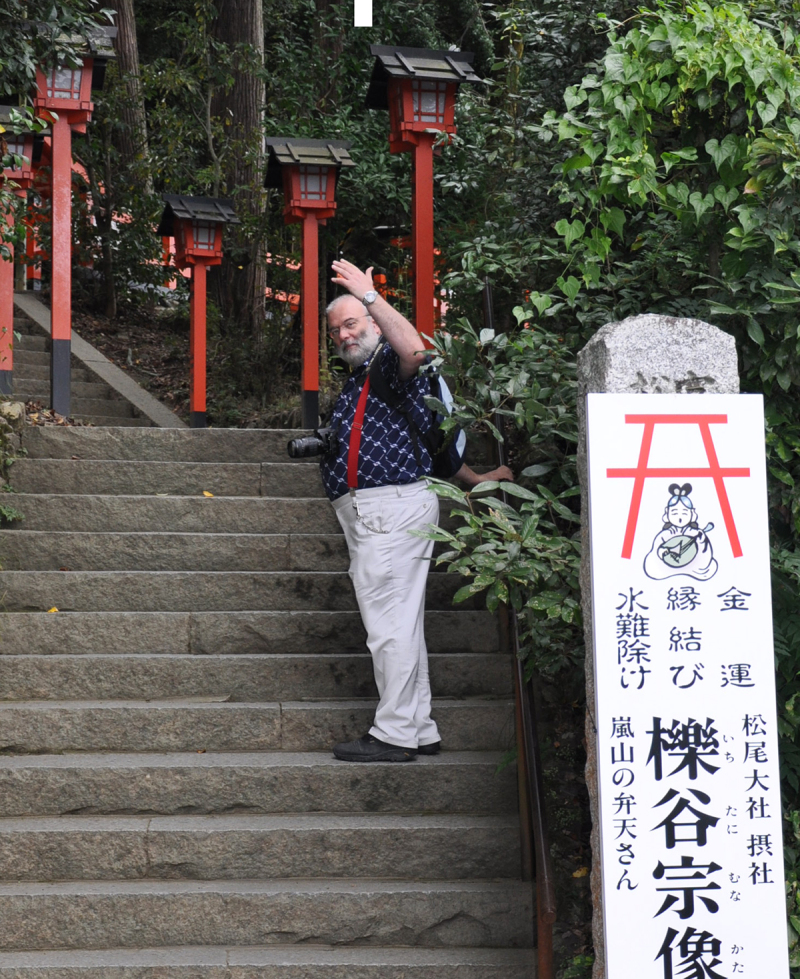
Pawel Liberski in Japan.

**Figure 4.**
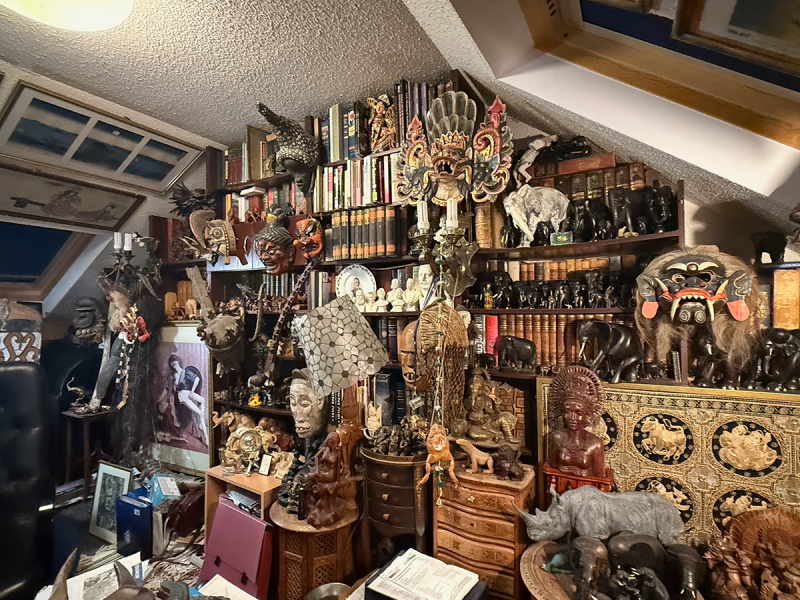
Pawel Liberski’s office at home (courtesy of Professor Maria Respondek-Liberska).

After graduating from the Medical Academy in Lodz, he earned his PhD and completed speciality training in neurology, followed by neuropathology. But even as a student, he was already fascinated by an exceptionally rare group of diseases – transmissible spongiform encephalopathies – at a time when few people had heard of them. This was long before Stanley Prusiner coined the term ‘prion’, and the name “prion diseases was introduced. During his first fellowship in Vienna while still a medical student, he drew attention not only for his striking appearance for a young trainee (a long beard and black Lennon-style glasses) but also for his intense passion for TSEs and electron microscopy. Those two passions stayed with him for his entire life. Professor Herbert Budka – at the time a young physician who supervised him during his Austrian fellowship – was initially taken aback by Pawel’s appearance and personality, yet he and Pawel became close friends in a short time. That friendship lasted to the very end, when Herbert Budka came to Lodz to accompany Pawel on his final journey.

Thanks to his passion and determination, after his studies he was able to go first to Edinburgh, to Alan Dickinson’s laboratory, and later to Carleton D. Gajdusek’s laboratory of CNS studies NIH, Bethesda. He formed many acquaintances and friendships then, and those friendships lasted until the end of his life. In November, at the Prion 2026 conference in Rio de Janeiro, Brazil, we remembered Pawel and his friend from Bethesda, Paul Brown, who had died shortly before him – without holding back our tears.

After returning to Poland, Pawel built a modern laboratory and gave many young people a real chance for scientific and professional growth. He was best known for his work on the ultrastructure of prion diseases, but his interests and contributions reached far beyond that – into autophagy, viral encephalitis, and tumours of the central nervous system. He authored authored more than 400 scientific papers and over 40 books; his works have been cited around 20,000 times, and his h-index stood at 47. He also served on the editorial boards of several international and Polish journals, including Prion.

Even though we live in a time when a scientist’s stature is often reduced to points and ‘productivity’, for me – and I hope for all of us – what matters most is not how many points someone accumulates, sometimes by publishing large numbers of minor papers, but what they truly achieved and how they were regarded by the international scientific community. The response from the prion field to Pawel’s death is the best proof of his visibility and charisma. It was deeply moving, and it also shows that in our field we are not only colleagues competing in science, but also good friends. I am truly grateful to Pawel for bringing me into this community.

I met Pawel in the late 1990s. He was already a young professor with achievements that were extraordinary by Polish standards at the time. Poland was only beginning to emerge from communism; equipment was hard to obtain, research funding was limited, and international networks were still taking shape. In those conditions, Pawel created a new, modern department at our Medical University of Lodz and established Poland’s Reference Center for Prion Diseases. When, in 2002, he offered me a position in his Department of Molecular Pathology and Neuropathology, I was truly delighted. He used to say to me – and to his other disciples – quoting Morpheus from The Matrix: ‘I can only show you the door. You’re the one that has to walk through it.’ I was fortunate to spend over twenty years working closely with him, standing by his side and supporting his scientific passions until the very end. He was an outstanding leader, and clearest proof of that was the way our laboratory reacted to the news of his death: no one tried to hide their tears. He died suddenly and far too early – on 19 August, at his home in Lodz – and, thankfully, his wife, Professor Maria Respondek-Liberska, was with him at home at that time.

Those fortunate enough to have known him understood that he was not only a colourful figure with a recognizable silhouette, usually dressed in eye-catching clothes, and a clever, inventive scholar, but also an exceptionally kind and generous friend-one who shared his knowledge, his contacts, and everything he had, perhaps with the exception of his food. I still remember once seeing him lift a snail from the road so it wouldn’t be crushed by a passing car.

Pawel, thank you for being with us.

